# Aqueous Dissolution and Recovery of Poly(Vinyl Alcohol) in Multilayer Film

**DOI:** 10.1002/cssc.202502750

**Published:** 2026-05-25

**Authors:** Pongkhun Prommart, Sixtus Nzeh, Pranabesh Sahoo, Hrushikesh Pujari, David O. Kazmer, Margaret J. Sobkowicz, Wan‐Ting Chen

**Affiliations:** ^1^ Department of Plastics Engineering University of Massachusetts Lowell Lowell Massachusetts USA

**Keywords:** dissolution, end‐of‐life, multilayer packaging, precipitation, solvent‐based recycling

## Abstract

In this work, solvent‐based recycling was applied to a multilayer film of PBAT‐PLA and PVOH, where the dissolution conditions were optimized and the reusability of the recovered PVOH was explored. The use of PBAT‐PLA blends provides the possibility of biodegradation, in addition to mechanical recycling. PVOH is used due to its ability to dissolve in water, eliminating the need for toxic solvents in dissolution recycling, and its high barrier properties. The temperature, time, film size, and stirring were explored and optimized for film dissolution in water. After dissolution, the reusability of the recovered PVOH material was explored by comparing its chemical and mechanical properties to those of virgin PVOH material. Stirring experiments showed that films of different sizes dissolve at the same rate with efficient stirring, while smaller film sizes dissolve faster than larger films in the absence of stirring. Characterization of virgin and recovered PVOH showed that the chemical structure of PVOH is mostly maintained after recovery. However, these results also indicate the loss of flexibility and thermal stability of the material due to the loss of important additives.

## Graphical abstract

Schematic of a water‐based recycling process for PVOH/PBAT‐PLA tri‐layer films, showing selective PVOH dissolution, process condition screening, and recovery of both polymer fractions.

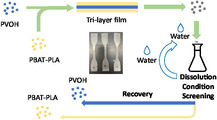

## 1 Introduction

Multilayer thermoplastic films are versatile since their multilayer structure allows for the combination of many functional properties for a wide range of applications. For that reason, multilayer films are a large portion of flexible packaging, accounting for approximately 26% of film consumption, with more than 100 million tons being manufactured each year [1]. However, it is difficult to separate the materials from multilayer films, which prevents their mechanical recycling at large scales and leaves most of them to be incinerated or landfilled [2].

Solvent‐based recycling is suitable for multilayer film due to the ability to separate and recover multiple materials by relying on the different solubilities of the materials. In other words, dissolution has been successfully applied to multi‐material plastic products and multilayer films. Sequential dissolution is where each dissolution step removes one polymer from the multi‐material product or film. After the target polymer has dissolved, the mixture is filtered to remove the undissolved fraction that can be further dissolved if needed. After filtration, the dissolved polymer can then be recovered, generally through the use of an antisolvent. Research on sequential dissolution has demonstrated efficient separation and the reuse of the materials for the same applications [3, 4]. In addition to the ability to handle mixed materials, solvent‐based recycling has been shown to effectively remove additives. For example, solvent‐based recycling was able to recover polypropylene from disposable face masks and remove significant amounts of blue colorants [5]. Solvent‐based recycling has also been used to remove significant amounts of plasticizers and additives in the recovery of polyethylene from multilayer film [3]. Many works have also shown that the chemical structure of polymers recovered through solvent‐based recycling is mostly maintained [4, 6, 7]; however, dissolution at elevated temperatures can result in thermal degradation. Additionally, removal of additives may result in the loss of functional properties or degradation during subsequent processing of the material. Several works have shown a slight decrease in yield stress of the material after solvent‐based recycling [6, 8]. Solvent‐based recycling has been shown to remove plasticizers and heat stabilizers, both of which are vital to the performance and processibility of the material. As a result, many researchers have observed a loss in flexibility and increased thermal degradation when processing materials recovered from solvent‐based recycling [6, 8]. After recovery, plasticizers and other additives can be added to achieve the desired properties. In the case of PVOH, plasticizers can be reintroduced through dry‐blending or directly into the extruder during processing [9, 10]. Alternatively, plasticizers can be blended into PVOH aqueous solution, making this process easily incorporated into solvent‐based recycling [11].

PVOH was first synthesized through the hydrolysis of poly(vinyl acetate) and was initially used for textile sizing in place of starch‐based sizes; this remained the largest application of PVOH until today [12, 13]. Due to PVOH’s water solubility and its ability to form film from solution, many of its other applications have been coatings, emulsifiers, and thickening agents [14, 15]. PVOH has also been used in packaging, particularly as coatings on paper or polyolefin films to provide barrier properties and in water‐soluble detergent packaging films [17, 18]. Though PVOH has been reported to have excellent oxygen barrier properties, this is only true in its dry state. Therefore, reported applications for PVOH as an oxygen barrier layer often require protection from moisture through crosslinking, blending with other materials, or encapsulation with a hydrophobic material [19, 20, 21].

Advances in PVOH formulation have allowed PVOH to be processed as a thermoplastic and successfully incorporated into multilayer film through conventional processing [23]. Recent studies have also demonstrated the incorporation of PVOH into multilayer structures with biodegradable polymers such as PLA and PBAT for packaging applications, where PVOH functions as an oxygen barrier layer while hydrophobic biodegradable outer layers provide moisture resistance to the film [21, 24, 25]. One of these studies showed that the incorporation of a PLA outer layer can reduce the equilibrium moisture content of the inner PVOH layer by approximately 50%, allowing PVOH to provide a significant oxygen transmission rate (OTR) reduction when compared to monolayer PLA film [24].

PVOH’s water solubility provides potential for dissolution‐based recycling with water as a solvent. This avoids the use of toxic solvents typically used in dissolution‐based recycling of other common plastics, such as xylene and toluene for dissolving polyethylene or benzyl alcohol and dichloromethane for dissolving polyethylene terephthalate [4]. Alternative end‐of‐life strategies for PVOH include mechanical recycling, anaerobic degradation, or composting. Mechanical recycling remains the most economical and environmentally friendly approach, but struggles to deal with multilayer films and can result in polymer degradation and downcycling, limiting material performance over multiple cycles [26, 27]. The environmental impact of composting and anaerobic degradation of PVOH has been explored. However, specific conditions are required to fully decompose PVOH through composting or anaerobic degradation, and their environmental performance depends strongly on process efficiency and life cycle assessment (LCA) assumptions [28, 29].

The objective of this work is to evaluate the feasibility of solvent‐based recycling for PVOH, a widely used water‐soluble polymer, and the effect of solvent‐based recycling on the properties and processibility of the material. First, this study focuses on optimizing the dissolution conditions that enable efficient recovery of PVOH from a multilayer structure. The temperature, time, film size, and stirring speed were explored for their influence on PVOH dissolution. Following dissolution, the recovered PVOH was subjected to chemical and mechanical characterization to assess changes in chemical structure, processibility, and performance. Thermal properties were analyzed using differential scanning calorimetry (DSC) and thermogravimetric analysis (TGA), while chemical changes were examined through Fourier‐transform infrared spectroscopy (FTIR), proton nuclear magnetic resonance (^1^H‐NMR), and gel permeation chromatography (GPC). Mechanical and functional performance was evaluated using tensile testing, OTR measurements, and melt flow index (MFI) analysis. By comparing properties before and after dissolution, this work aims to determine how solvent‐based recycling affects the structure, functionality, and processibility of PVOH.

## 2 Experiments and Methods

### 2.1 Feedstock

The poly(vinyl alcohol) resin used was Hydropol 33104P supplied by Aquapak (Birmingham, UK). The biodegradable material used for the multilayer film was Ecovio F2341, supplied by BASF (Ludwigshafen, Germany), which is a blend of PBAT and PLA. Both materials were received in pellet form and desiccant‐dried in an oven before use according to the suppliers’ recommendation. Ecovio F2341 was dried for 4 h at 70°C, and Hydropol 33104P was dried for 12 h at 50°C.

### 2.2 Processing Conditions

**FIGURE 1:**
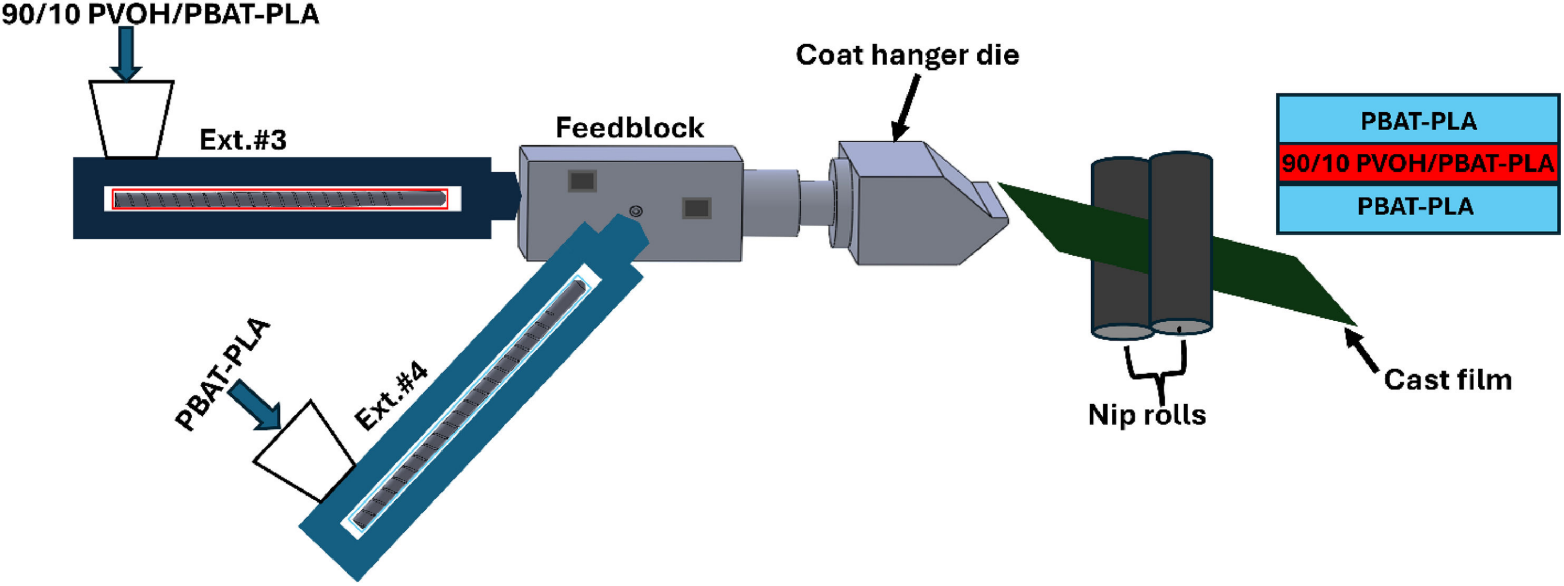
Coextrusion setup (adapted from literature [30]).

Tri‐layer films of PBAT‐PLA and PVOH were coextruded with two Teachline E20 single screw extruders (Dr. Collin, Maitenbeth, Germany) with a 20 mm screw diameter and an L/D ratio of 25:1. The core layer consisted of a solid blend of PVOH and PBAT‐PLA at a 90/10 weight ratio – blending PBAT‐PLA with PVOH provided significant plasticization and aided in the coextrusion of the two materials – while the skin layers consisted of 100% PBAT‐PLA. As illustrated in Figure 1, the two extruders are connected to a feedblock that channels the polymer melt into a coat‐hanger die. Extruder #3 supplies melt to the core layer, while the melt from extruder #4 is split to form the upper and lower layers [30]. The coat‐hanger die is designed to produce a film with an initial thickness of 0.5 mm and a width of 246 mm. The film was subsequently drawn down to a final thickness of approximately 150 µm. Processing temperatures for the tri‐layer films are outlined in Table 1. PVOH and PBAT‐PLA were compounded with a twin‐screw extruder (KZW 15TW, Technovel Corp, Osaka, JP) with a 15 mm screw diameter and an L/D ratio of 60:1. Processing conditions for compounding are outlined in Table 1 as well.

**TABLE 1:** Processing conditions of PVOH/PBAT‐PLA multilayer film.

Extruder	Screw speed, rpm	Temperature, °C						
		Zone 1	Zone 2	Zone 3	Zone 4	Zone 5	Zone 6	Zone 7
#3	50	90	180	185	190	220	230	230
#4	30	100	200	230	230	230	230	230
Twin‐screw	117	100	190	215	215	215	220	225

### 2.3 Dissolution‐Precipitation of PVOH

Sequential dissolution screening was carried out to establish consistent recovery conditions for subsequent experiments. First, the dissolution temperature was screened by varying the temperature while fixing the maximum dissolution time at 2 h. The minimum temperature required to achieve complete dissolution of PVOH within this time was selected. After identifying this temperature, a second screening was performed to determine the minimum dissolution time required for complete dissolution of PVOH from the tri‐layer film. In this step, the dissolution time was varied while all other conditions were held constant, and the shortest time for complete dissolution was selected for the recovery procedure. This approach was used to define a practical laboratory dissolution condition while limiting unnecessary thermal exposure of the material, rather than determining a true process optimum.

The effect of film size on the dissolution rate of the tri‐layer film was studied at the selected dissolution temperature. Four different diameters – 1.11, 2.22, 3.01, and 5.08 cm – of circular films were prepared using a hollow punch set. The amount of PVOH dissolved was then determined at 15, 30, and 45 min.

The effect of stirring on PVOH dissolution rate in tri‐layer film was also studied by comparing the amount of PVOH dissolved without stirring for two circular films having diameters of 1.11 and 3.01 cm, after 2 h of dissolution time. A longer time was used to account for the lower dissolution rate without stirring.

Finally, PVOH from tri‐layer film was recovered through dissolution and solvent casting (in water) and compared with virgin 33104P. Dissolution of PVOH was performed at 70°C and 1 h, followed by filtration. The PVOH solution was cast in a glass container and left to evaporate at ambient conditions for 2 days to avoid the formation of voids, followed by further drying at 60°C under vacuum for 1 day. Preliminary study of PVOH drying showed that most of the moisture is removed after 24 h at this condition.

### 2.4 Characterization

#### 2.4.1 Scanning Electron Microscopy (SEM)

SEM (JSM 7401F, JOEL, Tokyo, Japan) analysis was completed after sputter coating with a Denton Vacuum Desk IV. The samples were coated with gold for 10 s at a vacuum level between 60 and 70 torr. The images were taken using secondary electron imaging settings at 5 kV.

#### 2.4.2 TGA

TGA was performed (TGA2, Mettler Toledo, Columbus, OH) under a nitrogen environment. Standard aluminum oxide pans were used with a sample size between 7 and 10 mg. Temperature ramp tests began at 50°C and increased to 550°C at a rate of 20°C/min. STARe Evaluation software (Mettler Toledo) was used for additional analysis.

TGA curve deconvolution was performed assuming a linear superposition of PVOH and PBAT‐PLA. The mass fraction of each component was obtained by minimizing the least‐squared error between the measured curve and the reconstructed curve over the temperature range of 180°C and 550°C.

#### 2.4.3 DSC

DSC was performed (DSC 3+, Mettler Toledo, Columbus, OH). Samples of weight between 3 and 5 mg were analyzed in a 40 μl aluminum crucible under nitrogen (70 ml/min). Heat/cool/heat cycles were performed from 0°C to 250°C with a heating/cooling rate of 20°C/min.

#### 2.4.4 FTIR

FTIR spectra from 4000 to 400 cm^−1^ were collected by an attenuated total reflectance Fourier‐transform infrared spectrometer (ATR‐FTIR, Nicolet IS50, Thermo Scientific, Waltham, MA) coupled with Omnic software (version 7.3). A data interval of 1 cm^−1^ and a resolution of 4 cm^−1^ were used.

#### 2.4.5 GPC

GPC was used to determine the molecular weight of PVOH with a Waters Alliance 2695 HPLC system equipped with a PDA detector and a refractive index detector. Waters Styragel HR columns were used to determine the molecular weight distribution. The system used 100% tetrahydrofuran (THF) as the mobile phase at a flow rate of 0.9 mL/min. Each sample was dissolved in 100% THF at ambient conditions before being filtered through a 0.45 μm filter and analyzed at an injection rate of 10 to 20 mg/mL. Polyoxymethylene standards with molecular weights of 500 Da to 400 kDa were used to create the calibration curve. Three replicates were performed for each sample.

#### 2.4.6 Moisture Analyzer

The moisture content of 1 g PVOH was analyzed gravimetrically using COMPUTRAC Vapor Pro XL (Computrac, Richardson) moisture‐specific analyzer. Moisture was volatilized at 108°C until the end rate of 0.5 μg/s of moisture was reached. Five replicates were performed. Before the moisture content analysis using the moisture‐specific analyzer, both virgin PVOH and recovered PVOH were dried at 60°C for 54 h in a vacuum oven (AT19, Across International, Livingston, NJ) to ensure that their mass reached a constant and the adsorbed moisture was removed [31]. It is to be noted that this method of moisture analysis may not accurately reflect the true moisture content, since PVOH is likely to have tightly bound water that cannot be volatilized. For PVOH, Karl Fischer titration is the preferred method [32, 33]. This evaporation method only serves to determine the moisture content of PVOH samples relative to each other.

#### 2.4.7 ^1^H‐NMR

^1^H‐NMR was performed with a Jeol ECZ 400 system, operating at 400 MHz, to determine the hydroxyl content of PVOH. Spectroscopy measurements were carried out at 50°C with DMSO‐d_6_ solvent, a pulse width of 3 μs, a pulse delay of 4 s, and 160 scans to achieve a satisfactory signal‐to‐noise ratio.

The hydroxyl content is calculated by using Equation (1), adapted from literature [34].

(1) $$\begin{array}{c} \%\mathrm{OH}=\frac{\mathrm{OH}}{\mathrm{OH}+\frac{1}{3}\mathrm{Ac}}\times 100\% \end{array}$$

where Ac is the integral of the acetate methyl signal and OH is the integral of the hydroxyl signal.

#### 2.4.8 Pyrolysis Gas Chromatography Mass Spectrometry (Py‐GCMS)

Py‐GCMS (Agilent Technologies 7890B) equipped with an autosampler (AS 1020E, Frontier Laboratories Ltd., Fukushima, Japan) and metal capillary column (Ultra ALLOY‐5, 0.25 mm i.d. × 30 m × 0.25 μm immobilized polydimethylsiloxane film, Frontier Laboratories Ltd., Fukushima, Japan) was used to analyze virgin and recovered PVOH. PVOH sample of approximately 0.1 mg was tested using the double shot method, where the samples were subjected first to thermal desorption in the first stage at temperatures of 100°C–250°C (40°C/min), then fully pyrolyzed at 600°C. The system was set at a constant flow rate of 1 mL/min with helium carrier gas. An injection split ratio of 50:1 was used. The mass spectrometer (5977A, Agilent, Santa Clara, CA) was used in electron impact mode with an ionization energy of 69.9 eV and a scanning range of 30 to 800 m/z.

#### 2.4.9 MFI

The MFI was determined for virgin PVOH pellets and recovered PVOH using a Dynisco LMI5000 Melt Indexer (Dynisco, Franklin, MA) according to ASTM D1238. Testing was performed at 200°C with a load of 5 kg and a soak time of 6 min.

#### 2.4.10 Tensile Test

Tensile testing was performed on solvent‐cast films of virgin PVOH and recovered PVOH. Dissolution was performed at 70°C for 1 h. Approximately 0.3 mm thick films were cast into glass containers. The solution was left to evaporate at room temperature for 3 days, then further dried at 60°C for 1 day.

Tensile testing was performed according to ASTM D882 Standard Test Method for Tensile Properties of Thin Plastic Sheeting [35]. D882 type IV tensile bars were tested using an Instron 5966 tensile tester (Instron, Norwood, MA) at a crosshead speed of 50 mm/min. Five specimens were prepared and tested for virgin PVOH and recovered PVOH. The elastic modulus, yield stress, and yield strain were determined. Prior to testing, tensile specimens were conditioned at 50% relative humidity for at least 48 h per Section 8.1 of ASTM D882.

#### 2.4.11 Oxygen Transfer Rate Analysis

The OTR was determined according to ASTM D3985 on samples having an area of 5 cm^2^ for the solvent cast films of virgin PVOH and recovered PVOH (see sample preparation for tensile test) and carried out using MOCON OX‐TRAN 2/22 oxygen permeation analyzer (MOCON, Brooklyn Park, MN) [36]. Testing was performed at 23°C and 0% relative humidity.

#### 2.4.12 Statistical Analysis

Relevant data was analyzed using Microsoft Excel 2024. Graphs were generated using Origin Pro 2024. To assess statistical differences between pairs, Welch’s t‐tests were performed (*α* = 0.05). To assess overall differences among groups, one‐way ANOVA (*α* = 0.05) followed by pairwise comparisons using Welch’s t‐tests.

## 3 Results and Discussion

### 3.1 Dissolution of PVOH

#### 3.1.1 Screening of Dissolution Condition

**FIGURE 2:**
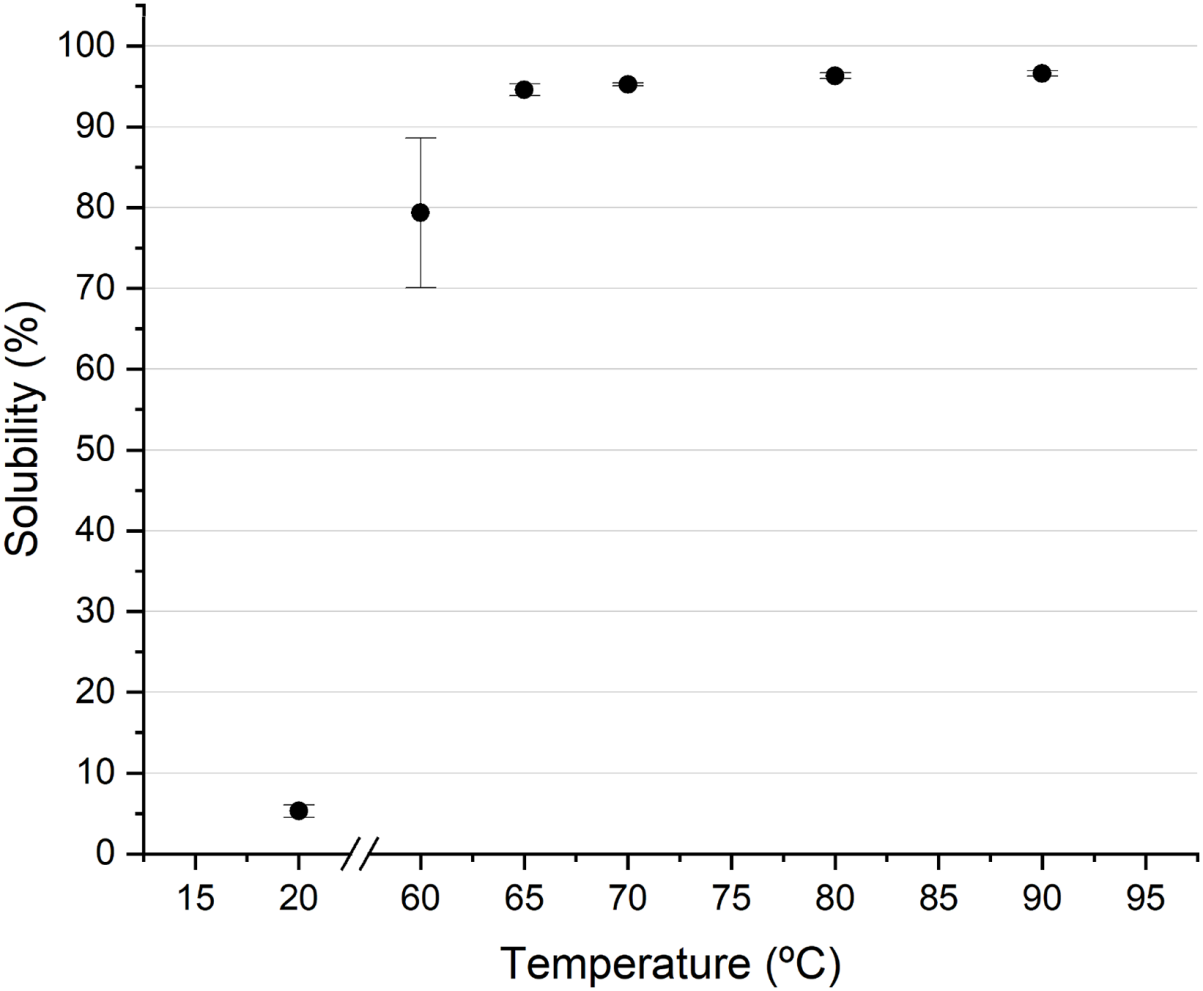
Solubility of PVOH 33104P versus temperature at 2 h.

Figure 2 shows the dissolution of PVOH in water at varying temperatures. At the temperature range tested, a thick gel layer was observed for PVOH, indicating that dissolution was occurring above the gel temperature and that PVOH was not fracturing. For dissolution at temperatures of 65°C and above, the solubility of PVOH remains relatively constant at around 96%. Lowering the dissolution temperature to 60°C, however, resulted in a significant reduction in solubility of PVOH to 79.3 ± 9.3%. At this temperature, the formation of a gel layer is observed. However, the dissolution rate is not high enough to achieve full dissolution. This observation aligns well with prior works that have reported an increase in erosion concentrations of polymer gels with decreasing temperature [37]. The shear rate generated through stirring was not sufficient to erode the gel layer, resulting in a sharp drop in the dissolution rate. The sharp drop in solubility below 65°C and a large standard deviation signify that dissolution below that temperature is not only too slow to achieve full dissolution, but that it is highly inconsistent.

**FIGURE 3:**
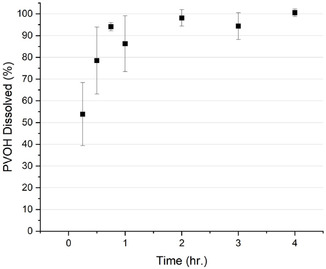
Percent PVOH dissolved in tri‐layer film (9 cm^2^) versus time at 70°C, 500 rpm.

Figure 3 shows the dissolution of PVOH/PBAT‐PLA tri‐layer films at 70°C at varying times. At 70°C, the majority of PVOH in the tri‐layer film is mostly dissolved by 1 h; this condition was selected for subsequent recovery of PVOH.

#### 3.1.2 Effect of Size and Stirring on Dissolution Rate

**FIGURE 4:**
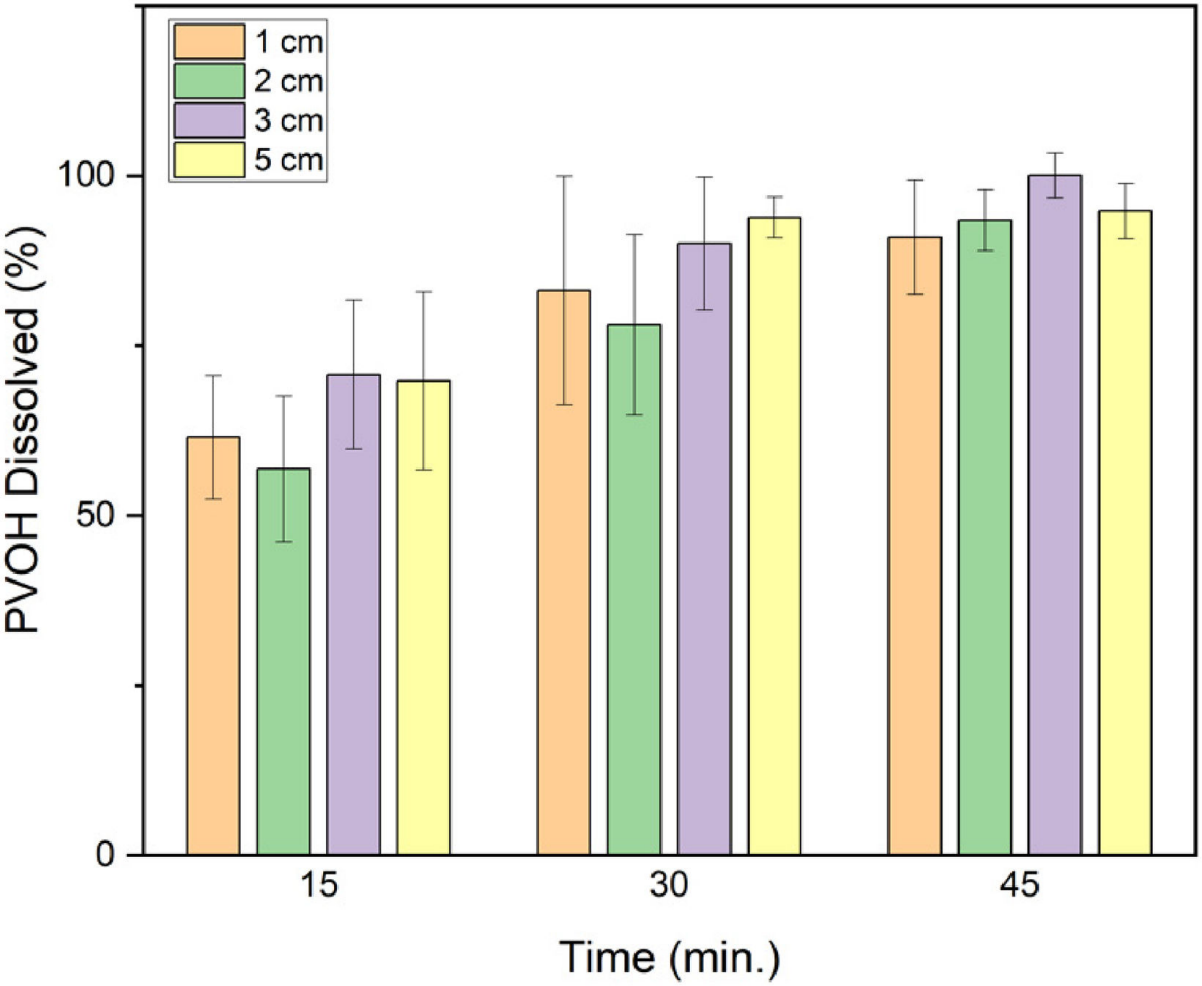
Percent PVOH dissolved in tri‐layer film of different sizes (1, 2, 3, 5 cm) versus time at 70°C, 500 rpm.

**FIGURE 5:**
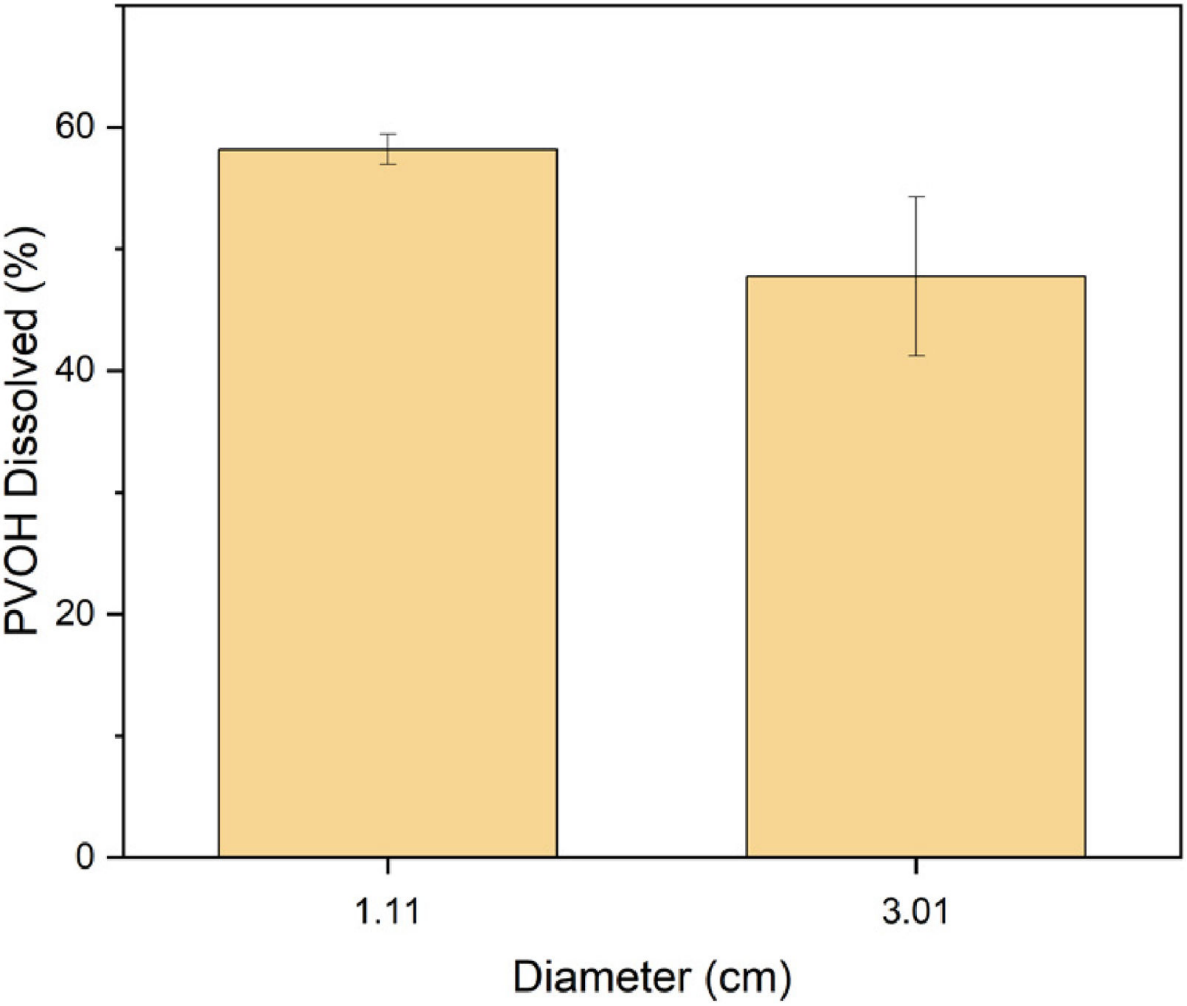
Percent PVOH dissolved in 1 and 3 cm tri‐layer film at 2 h without stirring.

To investigate the effect of film size and stirring on the dissolution rate of multilayer film, 4 different film sizes were dissolved at 70°C for 15, 30, and 45 min with stirring, shown in Figure 4, and two different film sizes were dissolved at the same temperature for 2 h without stirring, shown in Figure 5. Polymer dissolution is a two‐step process consisting of diffusion of the solvent followed by disentanglement at the corrosion concentration. With stirring, the similar dissolution rates observed across different film sizes and significant delamination suggest that dissolution was not controlled by diffusion through the exposed film edges. In the intact multilayer structure, access of water to the PVOH layer is initially limited to the film edges. However, hydrodynamic effects associated with agitation promote interfacial weakening and delamination of the tri‐layer structure, which exposes the PVOH layer. Once delaminated, the effective exposed PVOH surface area increases, and the surface area‐to‐volume ratio of the PVOH thin layers is similar across samples of different diameters. This leads to similar dissolution rates. Without stirring, full delamination was not observed, and PVOH is only accessible primarily at the film edges. Dissolution becomes geometry‐dependent and proceeds more slowly, with larger samples exhibiting reduced dissolution rates due to the reliance on the exposed edge for water diffusion.

### 3.2 Recovery of PVOH

#### 3.2.1 SEM

**FIGURE 6:**
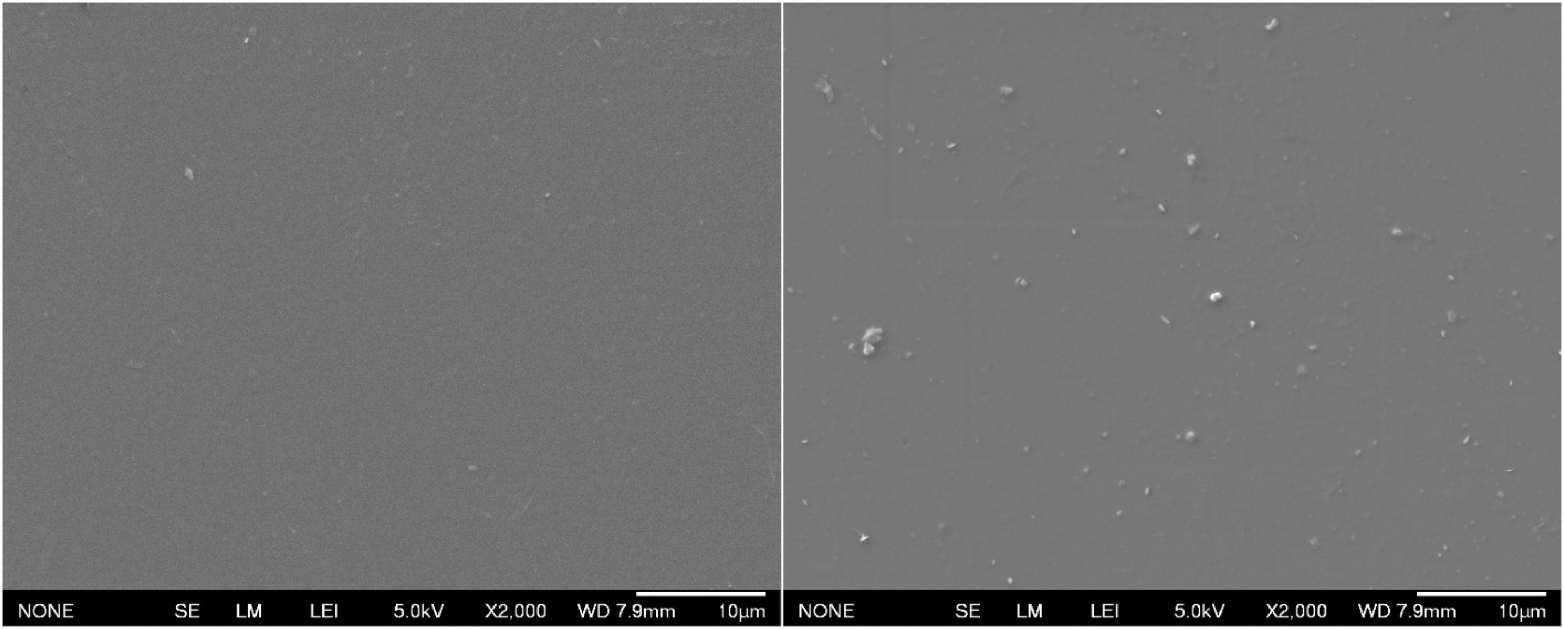
Surface SEM of vPVOH (left) and rPVOH (right).

SEM images of PVOH films at 2000x magnification, shown in Figure 6, reveal notable differences in surface morphology between the samples. Virgin PVOH film surface is smooth with minimal features. Recovered PVOH sample shows dispersed particles distributed across the surface on the order of a few micrometers. These particles are likely dispersed PBAT‐PLA that has passed through the filter paper (5 to 10 μm particle retention) during recovery.

#### 3.2.2 TGA

Table 2 shows the onset temperature, peak decomposition temperature, and the percent residue of virgin PVOH and PVOH recovered through the dissolution and drying process. Virgin PVOH showed higher onset temperature and peak decomposition temperature, signifying its superior thermal stability when compared to recovered PVOH. Both materials have comparable residue. However, the large standard deviation in the residue of the recovered material shows the variability in the recovery process. Similar findings in the literature suggest that the decrease in thermal stability may be due to the removal of heat stabilizers during the dissolution process [6].

**TABLE 2:** TGA results of 33104P and recovered 33104P (*n* = 3).

Material	***T***_**Onset**_ **, °C**	***T***_**Decomp**_ **, °C**	Residue, %
vPVOH	292.6 ± 1.0	332.6 ± 0.7	4.8 ± 0.6
rPVOH	276.1 ± 1.0	306.6 ± 9.0	4.6 ± 4.6

**FIGURE 7:**
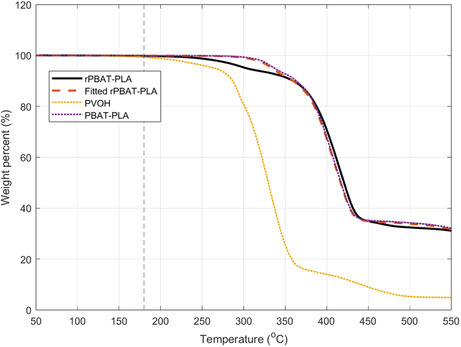
TGA deconvolution of recovered PBAT‐PLA (rPBAT‐PLA) from PVOH and PBAT‐PLA.

TGA was employed to evaluate the purity of recovered PBAT‐PLA (rPBAT‐PLA) after the removal of PVOH. Figure 7 shows the TGA curve for PVOH, PBAT‐PLA, and rPBAT‐PLA. After deconvolution of rPBAT‐PLA into PVOH and PBAT‐PLA, it was found that rPBAT‐PLA contained 1.20 ± 0.19 wt% of PVOH. The small amount of PVOH contamination in rPBAT‐PLA may be tolerable for enzymatic decomposition, but it could have negative effects on reprocessing. It is therefore important to dissolve and recover PBAT‐PLA as well to ensure high purity, as is often done in sequential dissolution processes [4].

#### 3.2.3 DSC

Table 3 shows the results obtained from DSC on the virgin PVOH and recovered PVOH. DSC thermograms of virgin and recovered PVOH are shown in Figures S1 and S2, and the raw data are included in Table S1 in the Supporting Information document. The crystallinity calculated from the second heating curve of the two materials is comparable. The recovered PVOH displayed a significant decrease in the peak melting temperature and crystallization temperature. The glass transition temperature, on the other hand, increased significantly after the recovery process. The increase in glass transition temperature and crystallization temperature indicates a reduction in chain mobility after recovery. Other works have observed a similar decrease in chain mobility and attributed this to the loss of plasticizers and small molecular weight polymer content, in addition to heat stabilizers [6, 38]. The reduction in chain mobility can also increase the melt viscosity, which can affect processibility and blend compatibility (note: more discussion is later presented in 3.2.3 MFI). Additionally, the reduction in the melting temperature signifies a reduction in the thermal stability of the material after the recovery process. Additional thermal degradation can occur during DSC, especially in the first heating curve. This degradation can result in a shift in the molecular weight distribution, slowing down the crystallization process [39, 40]. This observation aligns well with the decrease in thermal stability shown in the TGA results.

**TABLE 3:** DSC results of virgin PVOH and recovered PVOH (*n* = 3).

Material	***T***_**m**_ **, °C**	***T***_**c**_ **, °C**	***T***_**g**_ **, °C**	Crystallinity, %
vPVOH	194.8 ± 0.7	160.0 ± 2.5	27.4 ± 1.4	22.9 ± 1.9
rPVOH	189.2 ± 0.7	150.4 ± 4.4	39.4 ± 1.0	22.8 ± 2.1

#### 3.2.4 FTIR

The possible changes in the chemical structure due to the dissolution and recovery process were investigated through FTIR at three different spots in the films. Table 4 shows the FTIR characteristic peak positions of PVOH before and after recovery. FTIR spectra and characteristic peak raw data are included in Figure S7 and Table S3 in the supplementary information document. Table 4 shows that there were no significant shifts in the characteristic peak positions, indicating that the chemical structure of the polymer did not change significantly during the process.

**TABLE 4:** Characteristic peaks of virgin PVOH and recovered PVOH (*n* = 3).

Material	**Wavelength, cm** ^**−1**^			
	OH	CH3	C=O	C–O–C
vPVOH	3278 ± 6.3	2917 ± 0.6	1720 ± 9.6	1089 ± 0.5
rPVOH	3280 ± 4.1	2918 ± 1.6	1714 ± 2.0	1087 ± 1.1

#### 3.2.5 GPC

**FIGURE 8:**
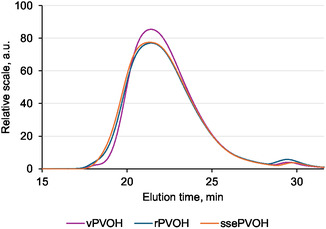
GPC chromatograms of vPVOH, rPVOH, and ssePVOH.

GPC was used to determine the effect of the dissolution and recovery process on the molecular weight and molecular weight distribution of the polymer. The average molecular weight and the molecular weight distribution can provide insights into the processibility of the material and whether any significant degradation has occurred [41]. To distinguish between the effects of extrusion and the effects of dissolution, samples of extruded pure PVOH (ssePVOH) were analyzed in addition to virgin and recovered PVOH. Figure 7 shows the chromatogram of the three samples, where two peaks can be observed at approximately around 21.5 and 29.5 min elution time, corresponding to the main polymer fraction and oligomer fraction, respectively. To quantify the oligomer fraction, a fixed elution time cutoff of 28.5 min was used to separate the two peaks across all samples (Figure 8).

Compared to virgin PVOH, the main polymer peak of recovered PVOH is broadened and shows an increase in the high molecular weight tail. A similar trend can be observed in extruded pure PVOH as well, suggesting that these changes are primarily an effect of extrusion rather than dissolution. Table 5 summarizes the number average molecular weight (Mn), weight average molecular weight (Mw), and polydispersity index (PDI) of the main polymer peak, along with the weight percentage of the oligomer fraction. Relative to virgin PVOH, recovered PVOH and extruded pure PVOH showed a slight increase in Mn and a more pronounced increase in Mw, indicating broadening towards higher molecular weights.

**TABLE 5:** Molecular weights and polydispersity index of virgin PVOH (vPVOH), recovered PVOH (rPVOH), and samples of extruded pure PVOH (ssePVOH) (*n* = 3).

Material	***M***_**n**_ **, kDa**	***M***_**w**_ **, kDa**	PDI
vPVOH	20.9 ± 0.3	93.7 ± 1.6	4.49 ± 0.05
rPVOH	21.2 ± 0.1	113.3 ± 0.4	5.35 ± 0.03
ssePVOH	21.7 ± 0.1	114.7 ± 1.1	5.29 ± 0.05

Effects from the presence of PBAT‐PLA in the recovered PVOH cannot be ignored. Transesterification reaction can occur, or the acidic decomposition product of PBAT‐PLA can catalyze crosslinking and degradation of PVOH, further contributing to the broadening of the molecular weight distribution [42]. However, the current data in this study do not allow these effects to be separated from the effect of thermal history. Overall, these results indicate that thermal processing of PVOH can lead to broadening of the molecular weight distribution due to competing mechanisms. Chain scission during processing generates low molecular weight species, while a small number of chains contribute to an increase in high molecular weight tail through crosslinking [40, 43].

#### 3.2.6 MFI and Moisture Content

The MFI was also measured to investigate the effect of dissolution and recovery on the processibility of the recovered PVOH. Table 6 shows the MFI of the virgin and recovered material. The moisture content is also reported, as water can act as a plasticizer in PVOH. It should be noted, as mentioned in the methods section, that the moisture content may be overestimated and only serves to represent relative differences. The recovered material exhibited a significant reduction of MFI from 1.431 ± 0.02 to 0.256 ± 0.05 g/10 min, more than fivefold decrease. In addition, the recovered material showed pronounced yellowing after the test, which is commonly associated with polyene formation as a result of thermal degradation of PVOH during melt processing [16]. This result aligns with findings from TGA, which indicates a reduction in thermal stability in the recovered PVOH.

**TABLE 6:** MFI and moisture content of virgin PVOH and recovered PVOH.

Material	MFI, g/10min	Moisture, %
vPVOH	1.431 ± 0.02	2.4
rPVOH	0.256 ± 0.05	1.6

The significant decrease in MFI is likely a result of the decreased chain mobility and reduction in thermal stability. While plasticizer loss is not directly measured in this study, previous work has reported the loss of low molecular weight species after solvent‐based recycling, which can further affect chain mobility and viscosity [44, 45].

#### 3.2.7 Tensile Strength

Tensile testing was performed on solution‐cast films for both the virgin and recovered material to observe the effect of the recovery process on the mechanical properties of the material. Table 7 shows the modulus, ultimate elongation, and ultimate stress. Recovered PVOH exhibited more than a fourfold increase in Young’s modulus and a significant reduction of ultimate elongation from 234.0 ± 50.5% to 150.5 ± 25.3%. Despite this loss in ductility, the ultimate stress of the material remains largely unchanged. The increase in stiffness and reduction in elongation indicate that the recovered material has reduced chain mobility. This behavior is consistent with molecular weight distribution changes observed in GPC and the increase in glass transition temperature observed in DSC.

**TABLE 7:** Modulus, elongation, and stress at break of virgin PVOH and recovered PVOH (*n* = 5).

Material	***ε***_**break**_ **, %**	***σ***_**break**_ **, MPa**	*E*, MPa
vPVOH	234.0 ± 50.5	50.3 ± 8.3	783 ± 133
rPVOH	150.5 ± 25.3	53.6 ± 9.1	3183 ± 630

#### 3.2.8 OTR

The OTR is an important property for films in food packaging applications. The OTR of the virgin and recovered PVOH is measured to probe the potential reuse of the material for the same or similar food packaging applications (Table 8). Generally, dry PVOH inherently provides low OTR due to the hydrogen bonding provided by the hydroxyl side groups and the crystalline nature of the material [16]. Therefore, any changes to the material’s ability to hydrogen bond or crystallize may affect the OTR. A significant increase in OTR was observed after recovery of the material. This may be attributed to the increase in small molecular weight fraction observed in GPC, which can disrupt the hydrogen bonding of PVOH or the packing of the chains, enhancing gas transport through the film [46].

**TABLE 8:** OTR of virgin and recovered 33104P (*n* > 3).

Material	**OTR, cc/m** ^**2**^ **‐day**
vPVOH	0.89 ± 0.64
rPVOH	3.50 ± 1.34

#### 3.2.9 ^1^H‐NMR

Alongside FTIR, ^1^H‐NMR was used to investigate the chemical structure of the polymer before and after recovery. ^1^H‐NMR was used to track the hydroxyl content of PVOH, summarized in Table 9, which has been shown to affect the material’s mechanical properties, processibility, and solubility [16]. In addition, the hydroxyl content of PVOH has been shown to be reduced as a result of thermal degradation [40]. The results show that the hydroxyl content of PVOH remained constant after processing, dissolution, and recovery.

**TABLE 9:** Hydroxyl content of virgin and recovered PVOH.

Material	%OH
vPVOH	96.4 ± 0.5
rPVOH	96.2 ± 0.7

#### 3.2.10 Pyrolysis Gas‐Chromatography Mass Spectrometer Analysis

The additives identified in PVOH and their corresponding GC–MS library match quality are listed in Table 10. The match quality of some plasticizers in recovered PVOH is slightly lower, not indicating a lower amount but a lower similarity between the library data and experimental results. A more in‐depth analysis is needed to better probe and quantify the amount of plasticizers remaining in the recovered PVOH. Glycerol, polyethylene glycol, dibenzyl sorbitol, and trimethylolpropane were identified as the plasticizers used in PVOH. Trimethylolpropane has been reported to be much less hygroscopic than glycerol [51]. Since the gas barrier property of PVOH is sensitive to moisture, trimethylolpropane can provide plasticization without sacrificing barrier property too heavily. Dibenzyl Sorbitol and trimethylolpropane are solid at room temperature, while glycerol is liquid at room temperature. Polyethylene glycol can exist as a viscous liquid or wax at room temperature, depending on the molecular weight. There are many advantages in using solid plasticizers for PVOH. Their higher melting and boiling points provide lower volatility, resulting in higher stability both in storage and during processing [51]. In addition, solid plasticizers provide less chain mobility at room temperature but melt during processing. This allows the material to have low melt viscosity, providing ease in melt processing, while maintaining high modulus when solid. Solid plasticizers can also act as a mild nucleating agent, providing clarity to the material [52]. However, the lower plasticizing effect of solid plasticizers may not always be desirable. It is advantageous in many cases to use a mixture of plasticizers to achieve a blend of their properties [51, 52]. This approach is most likely the case for this formulation of PVOH as well. Solvent‐based recycling has been shown to remove significant amounts of plasticizers that are not soluble in the solvent. In the case of this work, it is likely that dibenzyl sorbitol and polyethylene glycol have been removed during the filtration step due to their insolubility in water. It is therefore important to understand how to reintroduce these additives to provide melt‐processible PVOH after recovery through solvent‐based recycling.

**TABLE 10:** Additives identified in virgin and recovered PVOH.

Additive	Water solubility^a^	Function	Match quality, %	
			Virgin	Recovered
Glycerol	Soluble	Plasticizer	86	83
Polyethylene Glycol	Depends on Mw	Plasticizer	86	74
Dibenzyl Sorbitol	Insoluble	Plasticizer	92	95
Trimethylolpropane	Soluble	Plasticizer	85	76

a At room temperature (ref. [47, 48, 49]).

### 3.3 Insights and Discussion About Scaling Up Dissolution‐Precipitation of PVOH

There have been several attempts to scale up and commercialize solvent‐based recycling, such as Multi‐cycle by Fraunhofer Institute or Newcycling by APK AG. However, these processes have struggled to become commercialized [53, 54]. Results from this study, alongside several techno‐economic analyses and life cycle analyses comparing solvent‐based recycling to other end‐of‐life options, can give insights into the challenges of solvent‐based recycling.

Table 11 provides a summary of the four studies of Techno‐Economic Analysis (TEA) and LCA into solvent‐based recycling. Generally, these studies find that solvent‐based recycling provides both economic feasibility and lowered environmental impacts when compared to the production of virgin materials. However, these findings have been shown to depend strongly on the consistency and quality of the received recyclates and efficiency of the solvent recovery processes.

**TABLE 11:** Summary of TEA and LCA of solvent‐based recycling. Each letter indicates an analysis assumption.

Study	Process	Material	Major TEA findings	Major LCA findings	Scenario considered
A Novel Solvent‐Based Recycling Technology (Bar‐Ziv et al. 2024) [52]	STRAP (1,000–16,000 ton/yr)	Multilayer film of PE/EVOH/ PU/PET	STRAP can be profitable at modest scale	Very favorable: large GHG savings vs virgin resin (t ≥57% lower GHGs; in many scenarios >50% reduction). Results are sensitive to solvent recovery efficiency and electricity/steam assumptions.	a ^,^ c ^,^ d ^,^ f
			Solvent‐recovery efficiency is the most important economic factor.		
Evaluation of three solvent‐based recycling pathways for circular PP (Caudle et al. 2024) [53]	STRAP with three different recovery methods: Solvent–Antisolvent (SA), Temperature‐Swing (TS), and Supercritical Solvent (SS) (40,000 ton/yr)	PP	TS and SS more promising economically than SA.	CO_2_ emissions (cradle‐to‐gate): SA ∼1.30 kg CO_2_e/kg rPP, TS ∼0.92 kg CO_2_e/kg rPP, SS ∼0.32 kg CO_2_e/kg rPP.	a ^,^ c ^,^ d
			SA had worst economic performance due to antisolvent separation burdens.	Environmental competitiveness depends strongly on solvent choice, solvent recovery, and purity of incoming stream.	
The role of chemical and solvent‐based recycling within a sustainable circular economy for plastics (Klotz et al.) [54]	Solvent‐based recycling, depolymerization, pyrolysis, gasification (n/a)	PE, PET, PP, PS, PVC, ABS, HIPS, PA, PC, PUR	Large uncertainties — Profitability depends on scale, solvent recovery, and product markets; economics vary widely with yields, energy, solvent recovery.	Depolymerization/dissolution shows the lowest impact when operated well.	b ^,^ c ^,^ d ^,^ e ^,^ f
Toward a sustainable circular economy of multilayer films (Nazemi et al., 2025) [55]	STRAP vs downcycling, pyrolysis, incineration (3942 ton/yr)	Multilayer film of PE/PA	Downcycling (pelletizing) is more economically attractive. STRAP has higher capital/operating complexity. Economics depend on solvent recovery efficiency and recovered product quality/value. Sensitivity analysis shows high uncertainty.	STRAP outperforms incineration and often landfilling on GWP. But benefits decline when accounting for finite recovery cycles, imperfect solvent recovery, or degraded recovered polymer quality	b ^,^ c ^,^ d ^,^ f
Complementary roles for mechanical and solvent‐based recycling in low‐carbon circular PP (Nordahl et al. 2023) [56]	Mechanical recycling and solvent‐based recycling (n/a)	PP	Solvent‐based recycling increases market value but adds cost/energy Economics depend on scale and market for upgraded rPP.	Mechanical recycling yields the largest GHG savings (∼80% reduction vs virgin in studied cases); solvent‐based recycled PP still yields ∼30% GHG savings vs virgin.	b ^,^ c ^,^ d ^,^ e ^,^ f

a Cradle to gate.

b Cradle to grave.

c Sorting.

d Pre‐processing.

e Transportation.

f Closed‐loop credit.

Studies showed that the economic feasibility of a solvent‐based recycling plant depends heavily on the scale, where plants start to become economically viable at throughputs around 4,000 tons per year [55]. Excluding scale, many studies have shown that solvent recovery efficiency is the most important economic factor because of the high price of many solvents required to dissolve common plastics [56, 57]. The effect of solvent separation was observed in a study by Caudle et al., comparing three different pathways for solvent‐based recycling of polypropylene: solvent/antisolvent, temperature swing, and supercritical solvent [57]. In this study, the solvent/antisolvent recovery method required an extra separation step, driving up energy use in distillation, cost, and solvent losses. By contrast, the temperature‐swing process avoided antisolvent use and relied only on cooling to precipitate the polymer, lowering the energy requirement. The supercritical solvent process was able to recover most of the solvent due to the high volatility of propane. Due to the differences in separation efficiency, the solvent‐antisolvent process performed the worst economically. However, the temperature‐swing process and supercritical solvent process suffer from high vessel costs due to the extreme pressures involved.

The main drivers of environmental impact are the solvents, product quality, and energy usage. In addition to their high cost, the production of solvents can also require high energy usage. The separation efficiency is therefore equally important when considering the environmental impact of solvent‐based recycling. However, increasing the separation efficiency in distillation may require higher steam and electricity consumption, which can increase the environmental impact of the process.

Product quality plays a crucial role in determining the environmental impact of solvent‐based recycling. Many LCAs consider polymers recovered from solvent‐based recycling to be of virgin‐like quality and assume an infinite number of recycling iterations. However, the results from many studies, including this one, show some reduction in properties after recovery [6, 8]. The effect of recovered polymer quality can be observed clearly in the study performed by Nazemi et al., which showed that the global warming potential can increase significantly at a low number of recovery cycles [58].

In the case of solvent‐based recycling of PVOH, there is much room for improvement that can make the process more economical and environmentally friendly. Firstly, a higher PVOH concentration will be required for the process to be viable. A higher concentration will result in a lower amount of solvent used per unit polymer. With water as a solvent, the process may not be as sensitive to solvent use in terms of direct solvent emissions and cost when compared to more costly and toxic solvents. However, low PVOH concentrations will result in lower recovery rates, higher capital cost, higher operating cost, and higher environmental impact during separation. Secondly, a lower dissolution time for PVOH, which could be achieved at higher dissolution temperatures, is desirable to increase the output and lower the capital cost and operating cost. While the temperature selected in this study ensured complete dissolution within the defined timeframe, future work will systematically evaluate higher temperatures and shorter residence times to identify the optimal operating conditions that balance kinetics and polymer stability.

## 4 Conclusion

In this work, dissolution‐based recycling was applied to recover PVOH from PVOH/PBAT‐PLA tri‐layer film at 70ºC and 1 h of dissolution. PVOH was then recovered through filtration and solvent evaporation. DSC indicated high PVOH purity, and gravimetric measurements indicated that nearly 100% of PVOH was recovered through this method. In addition, particle size and stirring experiments showed that dissolution time is not strongly dependent on film size under efficient agitation.

Recovered PVOH showed a similar chemical structure to the virgin material, as shown by FTIR and ^1^H‐NMR, but exhibited lower thermal stability and significant losses in chain mobility. GPC indicated that chain scission and crosslinking are competing when PVOH is exposed to thermal history, and that the effect of dissolution and recovery on the degradation of PVOH is minor at this temperature. Nevertheless, a major increase in melt viscosity and rigidity due to effects from prior thermal processing can negatively impact the reprocessibility of the PVOH and prevent the use of the material for a similar application. Accordingly, post‐recovery characterization and compounding are required to ensure the properties and processibility of the recovered material.

## Author Contributions

**Pongkhun Prommart**, **Margaret J. Sobkowicz**, **David O. Kazmer** and **Wan‐Ting Chen**: conceptualization. **Pongkhun Prommart**, **Sixtus Nzeh**, **Pranabesh Sahoo**, **Hrushikesh Pujari**, and **Wan‐Ting Chen**:  methodology.  **Pongkhun Prommart**: validation.  **Pongkhun Prommart**: formal analysis. **Pongkhun Prommart** **,** **Pranabesh Sahoo** **,** **Margaret J. Sobkowicz** **,** **David O. Kazmer** **, and** **Wan‐Ting Chen** investigation.  **Pongkhun Prommart**: data curation.  **Pongkhun Prommart**: software validation. **Pongkhun Prommart**: writing‐original draft preparation. **Pongkhun Prommart**, **Sixtus Nzeh**, **Pranabesh Sahoo**, **Hrushikesh Pujari**, **David O. Kazmer**, **Margaret J. Sobkowicz**, and **Wan‐Ting Chen**: writing‐review and editing.  **Pongkhun Prommart**: visualization.  **Margaret J. Sobkowicz**, **David O. Kazmer**, and **Wan‐Ting Chen**: supervision.  **Margaret J. Sobkowicz**, **David O. Kazmer**, and **Wan‐Ting Chen**: project administration.  **Margaret J. Sobkowicz**, **David O. Kazmer**, and **Wan‐Ting Chen**: funding acquisition.

## Funding

This study was supported by Harnessing Emerging Research Opportunities to Empower Soldiers (HEROES) (Grant W911QY2020005 and PR2025‐2961), U.S. Department of Energy’s Office of Energy Efficiency and Renewable Energy (EERE), Advanced Materials & Manufacturing Technologies Office (AMMTO) (Grant DE‐EE0007897), and REMADE Institute, a division of Sustainable Manufacturing Innovation Alliance Corp. This report was prepared as an account of work sponsored by an agency of the United States Government. Neither the United States Government nor any agency thereof, nor any of their employees, makes any warranty, express or implied, or assumes any legal liability or responsibility for the accuracy, completeness, or usefulness of any information, apparatus, product, or process disclosed, or represents that its use would not infringe privately owned rights. Reference herein to any specific commercial product, process, or service by trade name, trademark, manufacturer, or otherwise does not necessarily constitute or imply its endorsement, recommendation, or favoring by the United States Government or any agency thereof. The views and opinions of authors expressed herein do not necessarily state or reflect those of the United States Government or any agency thereof.

## Conflicts of Interest

The authors declare no conflicts of interest.

## Supporting information

Supplementary Material
