# Supplementary material for: Aqueous Dissolution and Recovery of Poly(Vinyl Alcohol) in Multilayer Film
Source: ChemSusChem. 2026 May 25;19(10):e202502750. doi: 10.1002/cssc.202502750 (PMC13206348; doi:10.1002/cssc.202502750)
Supplement: Supplementary file 1 — Supplementary Material [file CSSC-19-e202502750-s001.pdf]

Supporting Information

Aqueous Dissolution and Recovery of Poly(vinyl alcohol) in Multilayer Film

Pongkhun Prommart, Sixtus Nzeh, Pranabesh Sahoo, Hrushikesh Pujari, David O. Kazmer, Margaret J. Sobkowicz, and Wan-Ting Chen*

Department of Plastics Engineering, University of Massachusetts Lowell, Lowell, MA, USA 01854

*Corresponding author: GraceWanTing_Chen@uml.edu

Number of Pages: 7

Number of Tables: 1

Number of Figures: 9

Table of Contents:

**Section 1**	DSC Thermograms
**Section 2**	TGA Thermograms
**Section 3**	Tensile Stress-Strain Plots
**Section 4**	FTIR Spectra
**Section 5**	Oxygen Transmission Rates
**Section 6**	1H-NMR Spectra

Section 1 DSC Thermograms

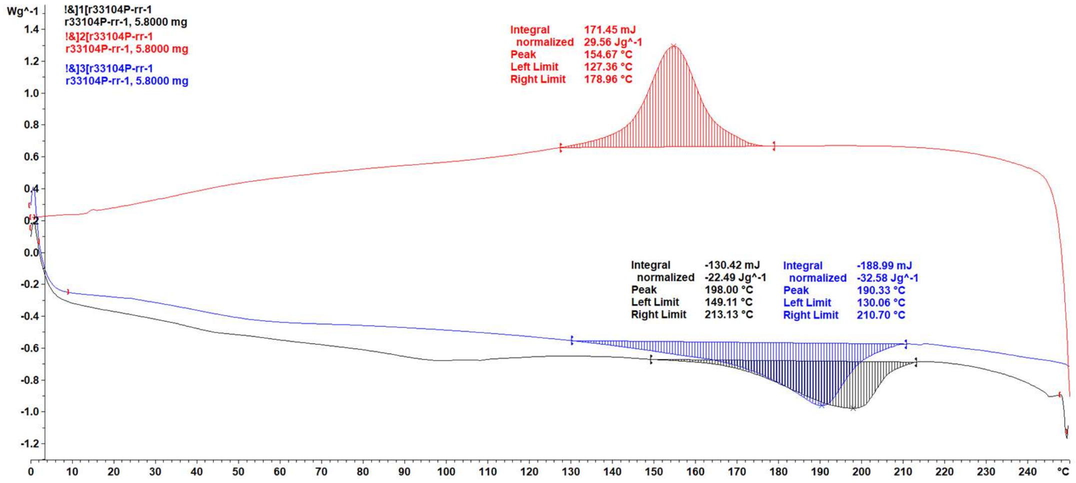

Figure S1. Virgin PVOH DSC curve.

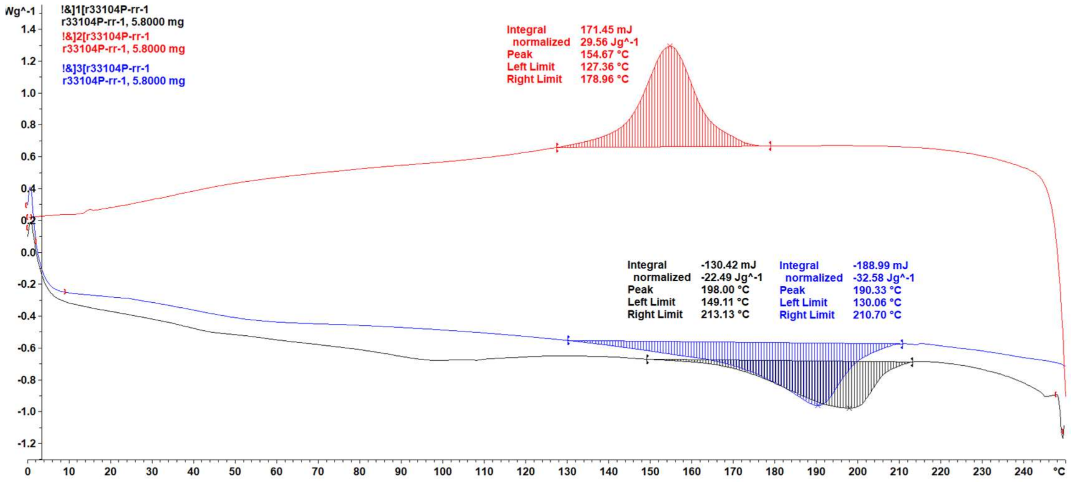

Figure S2. Recovered PVOH DSC curve.

Table S1. DSC raw data.

Sample	***Specimen***	T_m, °C	***Tc, °C***	***Tg, °C***	***Crystallinity, %***
vPVOH	1	195.1	158.7	28.4	20.3
vPVOH	2	194.1	159.7	26.5	21.5
vPVOH	3	195.4	163.4	27.3	23.4
rPVOH	1	190.0	155.0	38.3	25.0
rPVOH	2	188.7	150.0	39.9	20.9
rPVOH	3	188.8	146.3	39.9	22.6

Section 2 TGA Thermograms

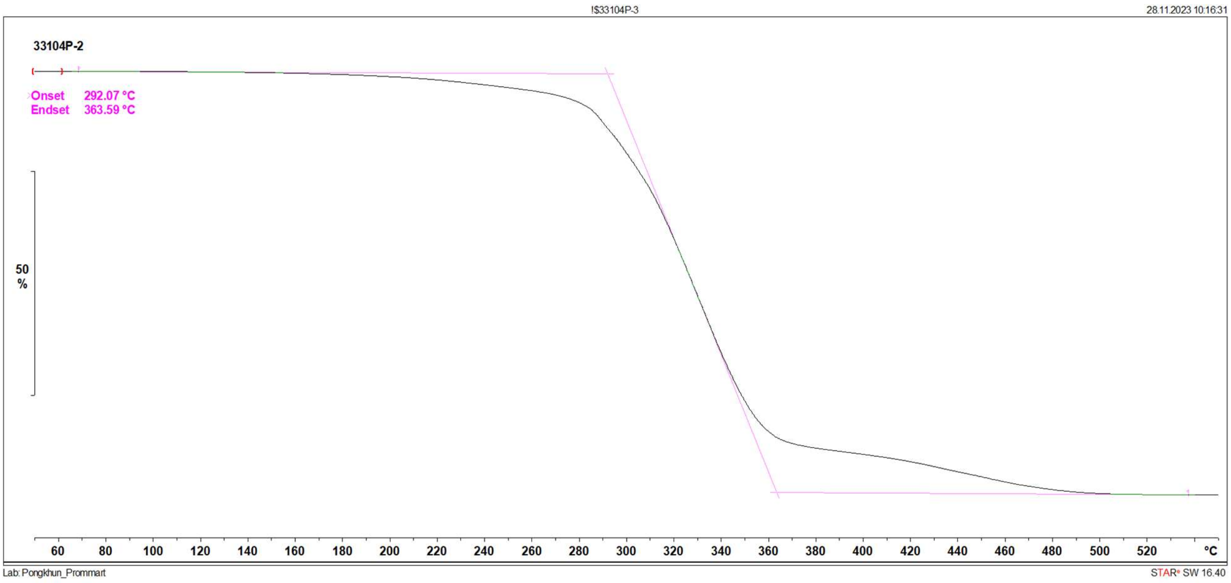

Figure S3. Virgin PVOH TGA curve.

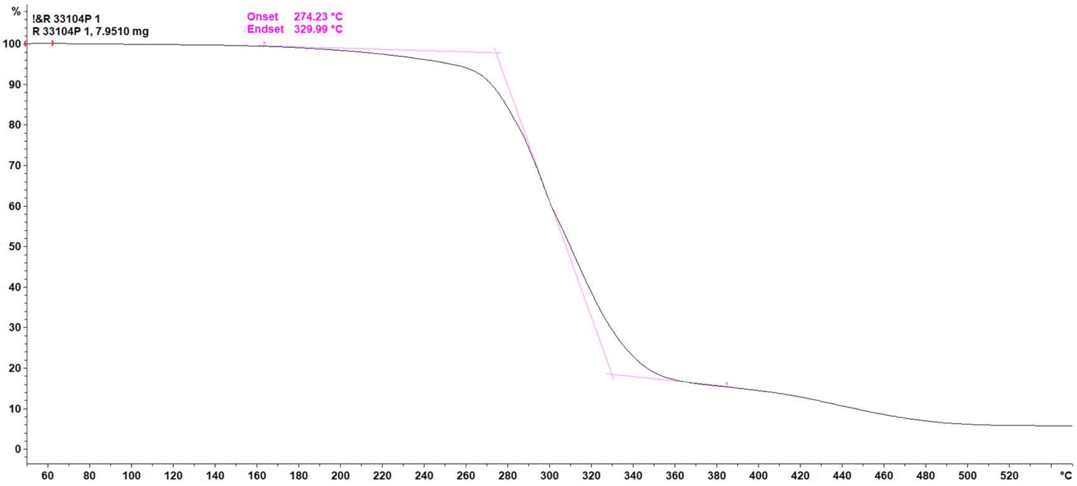

Figure S4. Recovered PVOH TGA curve.

Table S2. TGA raw data.

Material	***Specimen***	***T_{Onset}, °C***	***T_{Decomp}, °C***
vPVOH	1	292.1	332.9
vPVOH	2	292.1	331.8
vPVOH	3	293.7	333.1
rPVOH	1	275.7	296.3
rPVOH	2	277.2	313.3
rPVOH	3	275.3	310.0

Section 3 Tensile Stress-Strain Plots

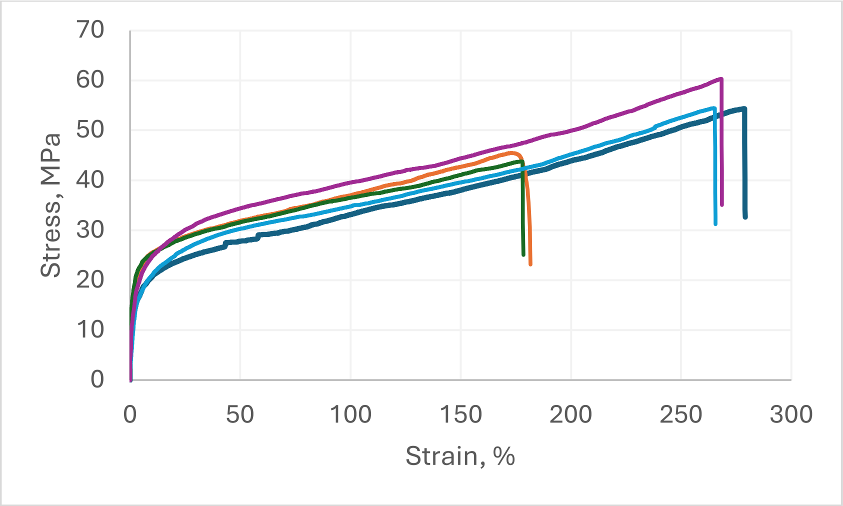

Figure S5. Tensile of virgin PVOH.

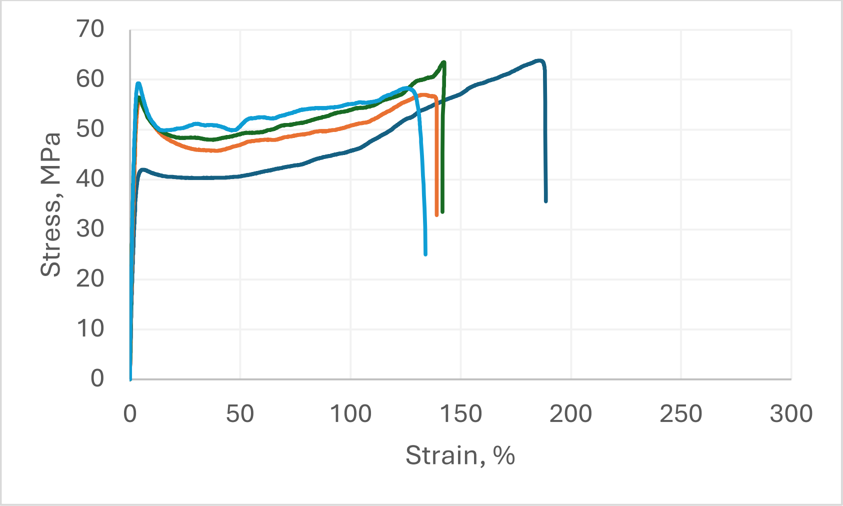

Figure S6. Tensile of recovered PVOH.

Section 4 FTIR Spectra

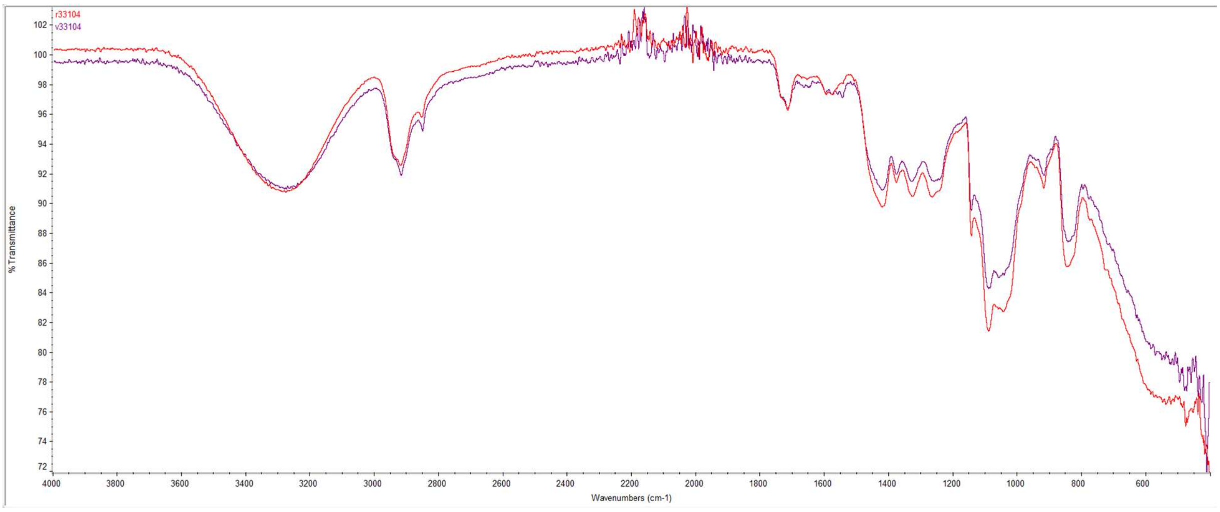

Figure S7. FTIR of virgin (purple) and recovered (red) PVOH.

Table S3. FTIR characteristic peaks raw data.

Sample	Specimen	OH	CH3	C=O	C-O-C
vPVOH	1	3284	2917	1731	1088
vPVOH	2	3280	2916	1716	1089
vPVOH	3	3272	2918	1713	1089
rPVOH	1	3284	2917	1712	1088
rPVOH	2	3279	2920	1716	1086
rPVOH	3	3276	2917	1713	1087

Section 5 Oxygen Transmission Rate

Table S4. Virgin and recovered PVOH OTR raw data.

Material	*OTR, cc/(m^2-day)*	*Thickness, mil*
r33104	1.06	6.0
r33104	1.619	12.0
r33104	0.03	4.5
r33104	3.232	11.0
v33104	0.865	6.5
v33104	0.803	8.5
v33104	1.613	5.5
v33104	0.039	4.5

Section 6 1H-NMR Spectra

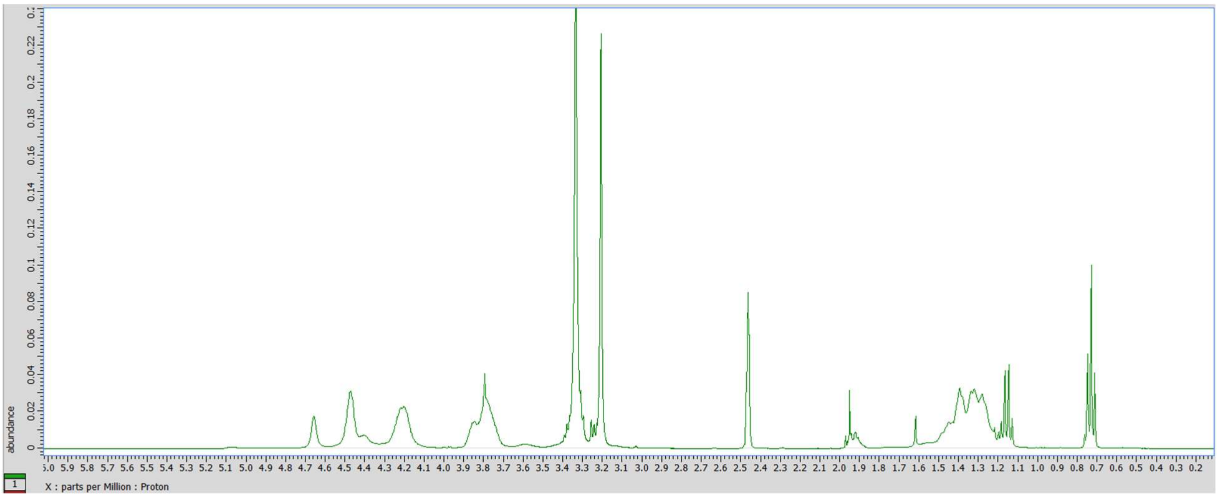

Figure S8. Virgin PVOH 1H-NMR spectra.

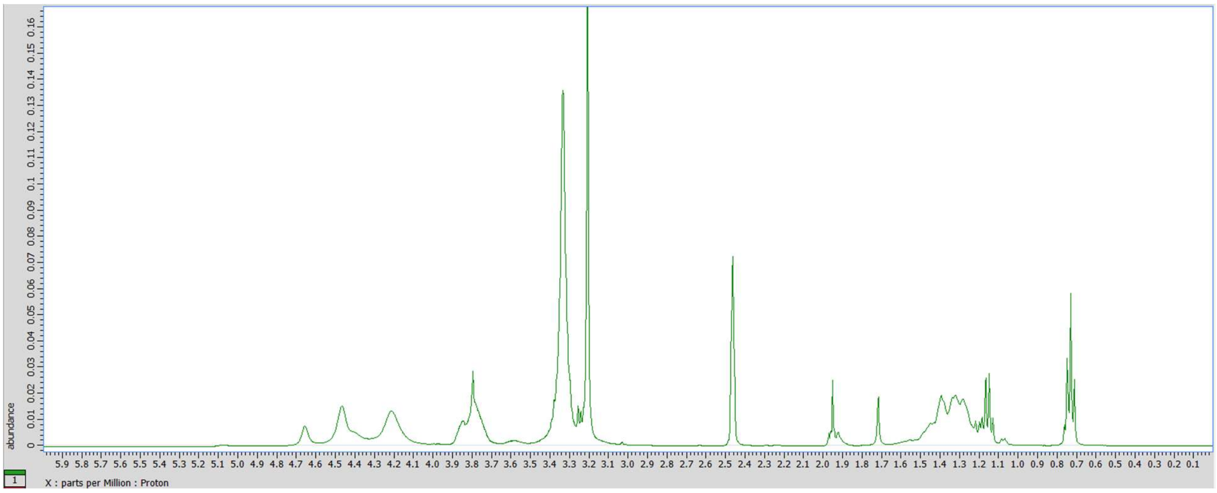

Figure S9. Recovered PVOH 1H-NMR spectra.
